# Pathologic and clinical correlates of region-specific brain GFAP in Alzheimer’s disease

**DOI:** 10.1007/s00401-024-02828-5

**Published:** 2024-11-24

**Authors:** Jared M. Phillips, Rebecca L. Winfree, Mabel Seto, Julie A. Schneider, David A. Bennett, Logan C. Dumitrescu, Timothy J. Hohman

**Affiliations:** 1 Vanderbilt Memory and Alzheimer’s Center, Vanderbilt University Medical Center, Nashville, TN USA; 2 Department of Pharmacology, Vanderbilt University School of Medicine, Nashville, TN USA; 3 Rush Alzheimer’s Disease Center, Rush University Medical Center, Chicago, IL USA; 4 Vanderbilt Genetics Institute, Vanderbilt University Medical Center, Nashville, TN USA

**Keywords:** Alzheimer’s disease, GFAP, Astrocytes, Transcriptomics, Proteomics, Biomarkers

## Abstract

Plasma glial fibrillary acidic protein (GFAP) is an emerging biomarker of Alzheimer’s disease (AD), with higher blood GFAP levels linked to faster cognitive decline, particularly among individuals with high brain amyloid burden. However, few studies have examined brain GFAP expression to clarify if peripheral associations reflect brain changes. This study aimed to correlate region-specific GFAP mRNA expression (*n* = 917) and protein abundance (n=386) with diverse neuropathological measures at autopsy in the Religious Orders Study and Rush Memory and Aging Project (ROS/MAP) and to characterize the interaction between brain GFAP and brain amyloid burden on downstream outcomes. We assessed GFAP gene expression in the dorsolateral prefrontal cortex, caudate nucleus, and posterior cingulate cortex with respect to core AD pathology (amyloid-β and tau), cerebrovascular (microinfarcts, macroinfarcts, and cerebral amyloid angiopathy [CAA]), proteinopathic (TDP-43, Lewy bodies), and cognitive outcomes. These associations were further examined at the protein level using tandem-mass tag proteomic measurements from the dorsolateral prefrontal cortex. We also assessed GFAP interactions with AD neuropathology on downstream outcomes. Cortical GFAP gene and protein expression were significantly upregulated in participants with a neuropathologically confirmed AD diagnosis at autopsy (all P_FDR_ < 3.5e−4), but not in individuals positive for tau pathology and negative for amyloid pathology (all P_FDR_ > 0.05). Higher cortical GFAP levels were associated with increased amyloid pathology, CAA pathology, and faster cognitive decline (all P_FDR_ < 3.3e−3). GFAP’s associations with phosphorylated tau burden and cognition were influenced by amyloid burden, being most pronounced among amyloid-positive individuals, confirming previous in vivo biomarker observations. No associations were observed between *GFAP* gene expression and outcomes in the caudate nucleus. Our results support previous biomarker findings and suggest that higher brain GFAP levels are associated with higher brain amyloid burden and faster cognitive decline among amyloid-positive individuals.

**Supplementary Information:**

The online version contains supplementary material available at 10.1007/s00401-024-02828-5.

## Introduction

Astrocyte reactivity plays a critical role in Alzheimer’s disease (AD) pathogenesis [13, 24, 26, 40]. Reactive astrocytes undergo extensive morphological and functional remodeling in response to a variety of central nervous system (CNS) insults, primarily serving to protect neural tissue and maintain homeostasis [13, 25, 46]. Specifically, in AD, reactive astrocytes release proinflammatory cytokines that increase the synthesis and release of neurotoxic amyloid-beta (Aβ) and reduce microglial Aβ phagocytosis [8, 21]. As such, astrocyte activation in response to early Aβ pathology initiates a positive-feedback loop that can persist potentially decades before symptom onset.

One highly conserved change in reactive astrocytes is strong upregulation of glial fibrillary acidic protein (GFAP), an intermediate filament protein expressed predominantly by astrocytes [52]. GFAP serves as a key cytoskeletal protein, helping astrocytes maintain their mechanical strength, support astrocyte–neuron interactions, and preserve blood–brain barrier integrity [52]. Increased GFAP largely reflects changes in the astrocyte cytoskeleton, including hypertrophy and extension of processes toward the site of injury [15].

Numerous studies have assessed the viability of elevated plasma GFAP as a biomarker for AD and amyloid pathology [1, 3, 11, 17, 37, 43, 49]. Plasma biomarkers are particularly useful due to their ease of collection and cost-effectiveness, and increasing effort is being applied to the evaluation of peripheral measurements of AD pathology and neurodegeneration [31]. Furthermore, recent findings leveraging *in vivo* measurements of peripheral GFAP found that high GFAP expression relates to worse cognitive outcomes, particularly in amyloid-positive individuals [3]. Despite these promising biomarker findings, very little is known about the association between GFAP levels in the brain and AD neuropathology, or whether upregulation of GFAP in the blood reflects upregulation in the brain, making the interpretation of plasma GFAP challenging. To date, few studies have examined how differences in GFAP abundance in the brain relates to AD neuropathology or concomitant pathways of injury in the brain. Similarly, it is not clear whether GFAP levels in the brain relate to antemortem cognitive performance, or whether such associations are modified by amyloid as is observed in the periphery. Characterizing such brain associations will shed light on the utility of GFAP as a biomarker of astrocytic changes in the AD brain.

To this end, we sought to interrogate the relationship between GFAP transcript expression and protein abundance in the brain with multiple AD-relevant outcomes, including amyloid and tau pathology as well as cognitive decline, while also assessing associations with concomitant pathways of injury. Leveraging data from the Religious Orders Study and Rush Memory and Aging Project, we evaluated GFAP expression in the dorsolateral prefrontal cortex (dlPFC), the posterior cingulate cortex (PCC), and the head of the caudate nucleus (CN) using bulk mRNA sequencing and GFAP protein abundance in dlPFC using tandem-mass tag mass spectrometry (TMT-MS). Furthermore, we tested for associations between GFAP and numerous non-AD pathologies, including infarcts, cerebral amyloid angiopathy, TDP-43, and Lewy bodies. Finally, we assessed interactions of GFAP and AD neuropathology on cognitive outcomes.

## Materials and methods

### Participants

We utilized autopsy and cognitive data from the Religious Orders Study (ROS) and the Rush Memory and Aging Project (MAP), collectively known as ROS/MAP, to conduct this study [7]. Data collection commenced in 1994 for ROS and in 1997 for MAP, resulting in extensive longitudinal clinical-pathologic data on aging and Alzheimer’s disease (AD) risk factors. ROS includes religious clergy members from across the United States, while MAP includes lay individuals from northeastern Illinois. Participants are older, free of known dementia at study initiation, and are primarily of European ancestry (see cohort demographics in Table 1). All participants consented to organ donation. Each study received approval from a Rush University Medical Center Institutional Review Board, including guidelines for data sharing under Institutional Review Board protocols. All participants provided informed and repository consents, along with an Anatomic Gift Act. Additionally, the analyses were approved by the Vanderbilt University Medical Center IRB.

**Table 1:** Participant demographics, ROS/MAP

	Dorsolateral prefrontal cortex bulk RNASeq	Caudate Nucleus bulk RNASeq	Posterior cingulate cortex bulk RNASeq	Dorsolateral prefrontal cortex TMT proteomics
Sample size	917	692	513	386
AD pathological diagnosis, no. (%)	554 (60)	423 (61)	299 (58)	221 (57)
AD clinical diagnosis, no. (%)	393 (43)	276 (40)	192 (37)	122 (32)
MCI clinical diagnosis, no. (%)	229 (25)	188 (27)	134 (26)	98 (25)
No cognitive impairment, no. (%)	295 (32)	228 (33)	187 (36)	166 (43)
*APOE*-ε4 carrier, no. (%)	234 (26)	187 (27)	130 (26)	79 (20)
Non-hispanic white, no. (%)	903 (98)	686 (99)	504 (98)	370 (96)
Female, no. (%)	597 (65)	450 (65)	318 (62)	271 (70)
Age at death (years)	89.42 ± 6.69	89.33 ± 6.46	89.34 ± 6.55	89.31 ± 6.51
Education (years)	16.38 ± 3.54	16.3 ± 3.54	16.38 ± 3.51	15.79 ± 3.55
Average follow-up time (years)	7.54 ± 4.97	7.66 ± 4.83	7.47 ± 4.86	8.75 ± 4.46
Global cognition score (last visit)	−0.8 ± 1.06	−0.78 ± 1.07	−0.7 ± 1.05	−0.51 ± 1.03
Post-mortem interval (hours)	7.56 ± 4.32	7.62 ± 4.39	7.02 ± 4.04	8.06 ± 5.46
CERAD, “moderate” or “frequent”, no. (%)	599 (65)	455 (66)	320 (62)	241 (62)
Braak III–VI, no. (%)	760 (83)	579 (84)	417 (81)	313 (81)

Values are mean ± standard deviation or number of samples (percent of the group)

Consortium to establish a registry for Alzheimer’s disease (CERAD) protocol for neuritic amyloid plaque density scores: (“none”, “sparse”, “moderate”, or “frequent”). Braak staging for neurofibrillary tangle distribution and severity (from 0; least severe, to VI; most severe)

*AD* Alzheimer’s disease, *MCI* mild cognitive impairment, *APOE-ε4* apolipoprotein E epsilon 4

### Genotyping

DNA was extracted from whole blood lymphocytes or frozen brain tissue, adhering to previously established quality control (QC) measures [30]. *APOE* genotyping was conducted by investigators blinded to cohort data at Polymorphic DNA Technologies. The *APOE* gene was sequenced to identify the isoforms *APOE*-ε2, *APOE*-ε3, and *APOE*-ε4, defined by codons 112 and 158 on exon 4.

### Neuropsychological composites

Details of the neuropsychological testing have been previously published [5, 6, 7] and additional documentation is available on the ROS/MAP website at www.radc.rush.edu. Briefly, 19 neuropsychological tests across five cognitive domains (episodic, semantic, and working memory, visuospatial ability/perceptual orientation, and perceptual speed) are used to calculate a composite global cognition variable in ROS/MAP. This variable represents a participant’s overall cognitive function. Raw scores from each test were converted to *z*-scores using the mean and standard deviation. The final composite score is derived by converting each test within each domain to a *z*-score and averaging all *z*-scores.

### Final summary clinical diagnosis

A clinical diagnosis was determined at each participant visit based on cognitive test scores, clinical judgment by a neuropsychologist, and diagnostic classification by a clinician (neurologist, geriatrician, or geriatric nurse practitioner) as described previously [5, 6, 7]. Clinical diagnoses of AD or other dementias followed criteria recommended by the joint working group of the National Institute of Neurological and Communicative Disorders and Stroke and the Alzheimer’s disease and Related Disorders Association (NINCDS/ADRDA). Diagnosis of mild cognitive impairment (MCI) was given to individuals judged to have cognitive impairment by the neuropsychologist but not meeting dementia criteria by the clinician. The final summary clinical diagnosis at the time of death was made by a neurologist, blinded to post-mortem data, based on a review of select clinical data from all years.

### Neuropathological measures

#### Core AD pathology

All neuropathological marker quantifications have been described previously [5, 6, 7]. Briefly, quantification of neuritic plaques and neurofibrillary tangles was based on silver staining of five brain regions (midfrontal cortex, midtemporal cortex, inferior parietal cortex, entorhinal cortex, and hippocampus) averaged to obtain a summary score of overall burden. Additionally, immunohistochemistry was used to calculate semi-quantitative scores for amyloid-β and phospho-tau abundance in the cortex, using antibodies specific to Aβ1-42 and abnormally phosphorylated tau (AT8 epitope), respectively, based on the average of eight regions (hippocampus, entorhinal cortex, midfrontal cortex, inferior temporal cortex, angular gyrus, calcarine cortex, anterior cingulate cortex, and superior frontal cortex).

#### Cerebrovascular pathology

Macro infarcts were visualized on fixed slabs and dissected for confirmation [2, 38]. Microinfarcts were examined on 6 µm paraffin-embedded sections, stained with hematoxylin/eosin. Gross and microinfarcts were categorized as present (1) or absent (0) based on visual inspection in nine brain regions (midfrontal, middle temporal, entorhinal, hippocampal, inferior parietal and anterior cingulate cortices, anterior basal ganglia, midbrain, and thalamus) [2]. A semi-quantitative score for cerebral amyloid angiopathy (CAA) was measured by amyloid-β immunostaining in neocortical regions (midfrontal, midtemporal, angular, and calcarine cortices), and was scored on a scale from 0 to 4 (0 = no pathology, 4 = severe pathology). A meningeal and parenchymal vessel score was obtained for each brain region, and the maximum of these was then used in each case. Final scores were averaged across regions [9].

#### TDP-43 and Lewy body pathology

TDP-43 immunohistochemistry was performed on eight brain regions using phosphorylated monoclonal TAR5P-1D3 TDP-43 antibody, and the presence of TDP-43 cytoplasmic inclusions in neuron and glia was assessed for each region [28]. A dichotomized variable representing no TDP-43 pathology or TDP-43 pathology in the amygdala only (0) and TDP-43 pathology extending beyond the amygdala (1) was leveraged in our analyses. Lewy body stages were determined by α-synuclein immunostain and encompassed four stages [39]. A dichotomized variable representing Lewy body pathology outside the neocortex (0) and neocortical-type (1) was used in our analyses.

### Autopsy measures of GFAP mRNA expression

As previously described [5], a standardized protocol for post-mortem biological specimens was used. RNA extraction from specific brain regions was conducted using a Qiagen miRNeasy mini kit along with an RNase-free DNase Set for quantification on a Nanodrop. The integrity and purity of the RNA were assessed using an Agilent Bioanalyzer. Samples with an RIN score greater than five were included for bulk next-generation RNA sequencing. RNASeq was generated in 946 participants.

Sequencing was performed in multiple phases. Phase one focused on the dorsolateral prefrontal cortex (dlPFC). Phase two added more dlPFC samples and included samples from the posterior cingulate cortex (PCC) and the head of the caudate nucleus (CN). Phase three included additional participant samples from the dlPFC. Detailed information on RNA processing and sequencing is available on Synapse (syn3388564).

In summary, phase one employed poly-A selection, strand-specific dUTP library preparation, and Illumina HiSeq with 101 bp paired-end reads, achieving a coverage of 150 million reads for the first 12 reference samples. These deeply sequenced reference samples included two males and two females from non-impaired, mild cognitive impairment, and Alzheimer’s disease cases. The remaining samples were sequenced with a coverage of 50 million reads. Phase two used the KAPA Stranded RNA-Seq Kit with RiboErase (kapabiosystems) for ribosomal depletion and fragmentation. Sequencing for this phase was performed on an Illumina NovaSeq6000 with 2 × 100 bp cycles, targeting 30 million reads per sample. In phase three, RNA was extracted with a Chemagic RNA tissue kit (Perkin Elmer, CMG-1212) using a Chemagic 360 instrument, and ribosomal RNA was depleted using RiboGold (Illumina, 20,020,599). Sequencing for phase three was carried out on an Illumina NovaSeq6000 with 40-50 million 2 × 150 bp paired-end reads.

Data processing and QC of RNA sequencing runs were performed by the Vanderbilt Memory and Alzheimer’s Center Computational Neurogenomics Team using an automated pipeline and are described in detail elsewhere [41, 50]. Samples whose last visit was >5 years before death or who had non-AD dementia were excluded. This quantification yielded measurements for GFAP in 917 samples. We further assessed correlations of GFAP protein and transcripts in the DLPFC with available cytokine/chemokine transcripts known to be upregulated in reactive astrocytes as reported by Sofroniew [45], yielding five inflammatory transcripts available for correlation analyses.

### Cellular fraction data

A deconvolution technique was previously employed to derive cellular fraction data for a subset of ROS/MAP participants [27]. This approach involved a subset of bulk RNA samples from the dlPFC which also had single-nucleus data (N = 48 individuals and 80,660 single-nucleus transcriptomes). These data were used to identify the best predictors of each cellular component (e.g., excitatory neurons, microglia, oligodendrocytes, etc.) by utilizing all genes in the RNAseq data to build models with the most optimized set of genes. The process of isolating and extracting nuclei from frozen tissue has been previously described [16]. In summary, the analysis of single-nucleus data (snRNA-seq) employed high-throughput droplet technology and massively parallel sequencing following the DroNc-seq protocol [15], with modifications for the 10X Genomics Chromium platform. Gene counts were obtained by aligning reads to the hg38 reference genome (GRCh38.p5) using CellRanger software. Unspliced nuclear transcripts were included by counting reads mapped to pre-mRNA. Each individual library was quantified for pre-mRNA and then aggregated to equalize read depth between libraries, generating a gene count matrix.

The quality control criteria for cell inclusion have been described in detail previously [27]. The final dataset comprised 17,926 genes in 75,060 nuclei. This snRNA-seq data were used in a regression-based approach to generate a reference expression profile and decompose bulk RNA sequencing data, resulting in cellular fraction estimates for each sample across eight cell types (microglia, astrocytes, inhibitory neurons, excitatory neurons, oligodendrocytes, oligodendrocyte progenitors, and endothelial or pericyte cells).

### Autopsy measures of GFAP protein expression

GFAP protein expression was quantified using isobaric tandem-mass tag mass spectrometry (TMT-MS) on dlPFC tissue from 400 ROS/MAP samples (syn17015098). Briefly, protein abundance was determined using brain tissue samples from the dorsolateral prefrontal cortices of 400 participants leveraging the UniProtKB human proteome database containing both Swiss-Prot and TrEMBL reference sequences (downloaded on 21 April 2015, processed data available at syn21266454) as reported previously [18]. Samples whose last visit was >5 years before death or who had non-AD dementia were excluded. This quantification yielded measurements for GFAP in 386 samples.

### Statistical analyses

Statistical analyses were conducted in R v4.1.2 using the R Studio IDE (https://www.rstudio.com/). Multiple linear regression models were employed for cross-sectional cognition and pathological outcomes to analyze the data, while linear mixed-effects models were used for longitudinal cognition. Models were executed separately for regional GFAP expression. We utilized generalized linear and proportional odds models for binomial and multinomial cerebrovascular outcome variables, respectively. Linear regression models adjusted for age at death, sex, and post-mortem interval. Models with cognition as the outcome also included education and the time in years between the final visit and death. In mixed-effects regression models, time was represented as years from the final visit, with both time and intercept included as fixed and random effects. Measurements of AD pathology through immunohistochemistry and silver staining were square root transformed to better approximate a normal distribution. Secondary analyses were performed to account for potential variation in model predictions due to astrocytic cell-type fraction by including this estimate as a covariate. Furthermore, in models assessing the interaction of GFAP expression and amyloid status, we leveraged a binary variable where amyloid negativity was defined as CERAD “none” or “sparse”, while amyloid positivity was defined as CERAD “moderate” or “frequent.” When assessing GFAP associations with non-AD pathologies, we also ran amyloid-stratified models using the binary amyloid status variable.

All models were corrected for multiple comparisons using the Benjamini and Hochberg (1995) false discovery rate based on the total number of tests completed, accounting for all GFAP predictors across modalities and outcomes (*N* = 66). Statistical significance was determined using the a priori threshold of *p* < 0.05. Among the 917 participants with *GFAP* mRNA measurement from the dlPFC, 668 participants also had *GFAP* measurement from the CN and 511 participants also had measurement from the PCC. There were 435 participants with *GFAP* measurements from all three brain regions. 281 participants had both mRNA transcript and protein measures of GFAP from the dlPFC.

## Results

Table 1 summarizes participant characteristics. Participants were long-lived (mean age at death > 89 years), predominantly of European ancestry (≥ 96%), female (≥ 62%), and highly educated (mean ≥ 15.8 years of education). The percentage of participants with greater severity and/or progression of neuropathology as measured by Braak staging or CERAD scoring was similar across brain regions.

### Cortical GFAP expression is upregulated in clinically and pathologically confirmed AD

**Fig. 1:**
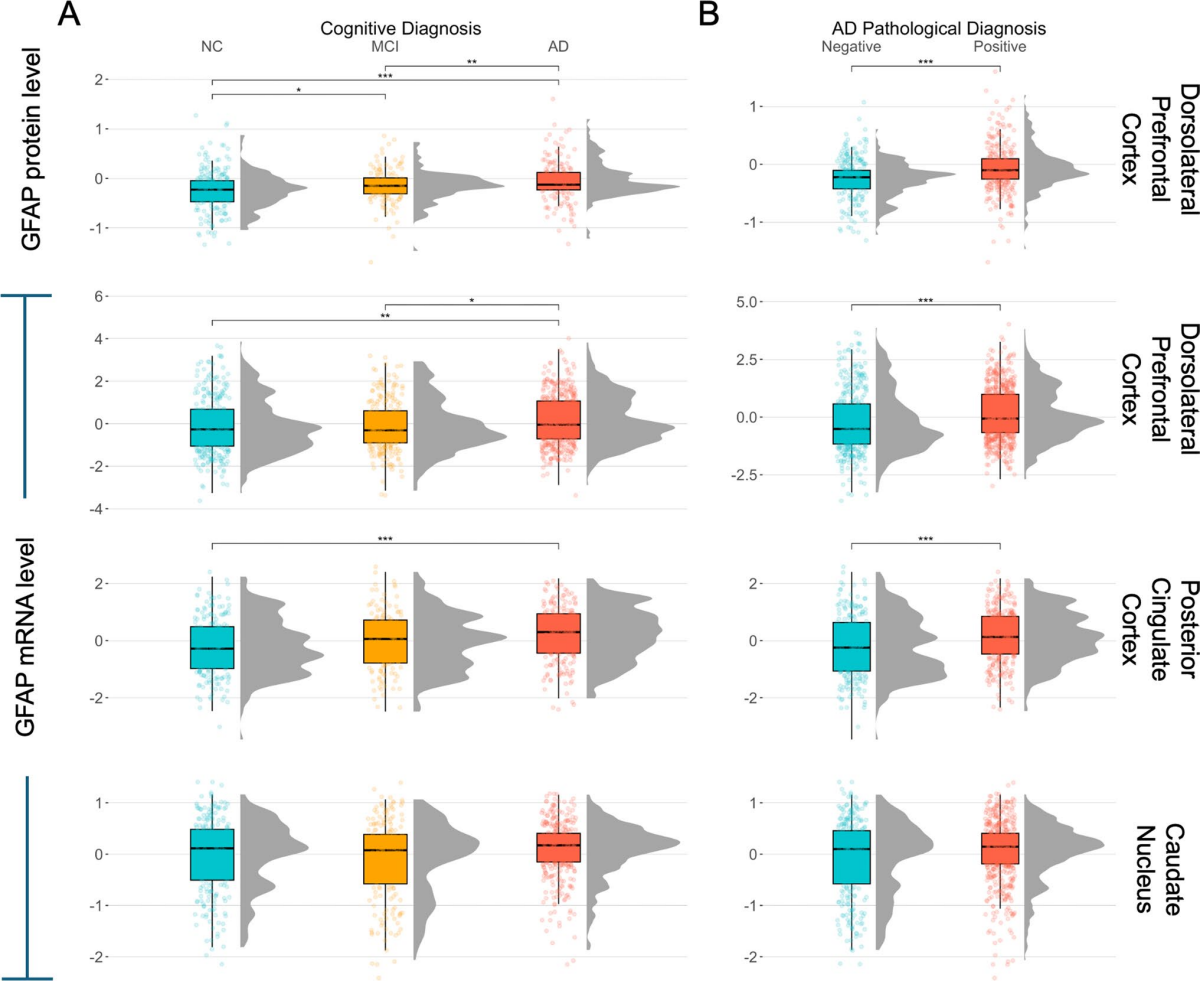
GFAP expression across diagnostic status. Cortical *GFAP* mRNA and protein are higher in those with a clinical or pathologic AD diagnosis than controls, while caudate expression values do not differ across diagnoses. **A** Final summary clinical diagnosis: normal cognition (NC), mild cognitive impairment (MCI), and Alzheimer’s disease (AD). **B** Pathologic diagnosis according to neuropathologic staging (CERAD and Braak) NIA-Reagan criteria. Positive: high or intermediate likelihood of AD and negative: low likelihood or no AD. Significance values are derived from pairwise Student t tests and FDR-corrected results. ns: *p* > 0.05. *p ≤ 0.05, **p ≤ 0.01, ***p ≤ 0.001

*GFAP* mRNA in the dlPFC and PCC was higher in individuals with a clinical or pathologic diagnosis of AD compared to those with normal cognition (NC) or no pathologic diagnosis (Fig. 1). Levels of *GFAP* mRNA in the CN did not differ across clinical or pathological diagnosis (Fig. 1). We further combined cognitive and pathological diagnoses and found that cortical GFAP transcript and protein levels are upregulated in participants with pathologically confirmed AD compared to cognitively unimpaired, pathology-negative individuals (Supplementary Fig. 1). Furthermore, *GFAP* mRNA in the dlPFC and PCC was highest in Aβ+/tau+ individuals, and interestingly, *GFAP* mRNA and protein expression in the dlPFC was significantly lower in Aβ-/tau+ individuals when compared to Aβ+/tau+ individuals (Supplementary Fig. 2). GFAP expression was positively correlated across brain regions and data types (DLPFC and CN r = 0.40, p = 4.2e-27; CN and PCC r = 0.45, p = 5.7e-24; DLPFC and PCC r = 0.67, p = 4.9e-67; DLPFC RNA and protein r = 0.25, p = 1.6e-5). As expected, *GFAP* mRNA was found to be correlated with the astrocytic cell-type fraction (r = 0.38, p = 2.2e-16). Finally, *GFAP* transcripts in the DLPFC were significantly, positively correlated with *CXCL1* (r = 0.2, p = 5.9e-10), *TGFB1* (r = 0.31, p = 8.9e-23), and *CXCL16* (r = 0.39, p = 3.3e-35). GFAP transcripts were negatively correlated with *IL11* (r = -0.2, p = 1.9e-9) and were not correlated with *CXCL12* (r = 0.03, p = 3.2e-1). GFAP protein levels were not significantly correlated with any of the inflammatory transcripts (r = -0.07 – 0.11, all p > 0.05).

### Cortical GFAP expression is associated with AD pathology

**Fig. 2:**
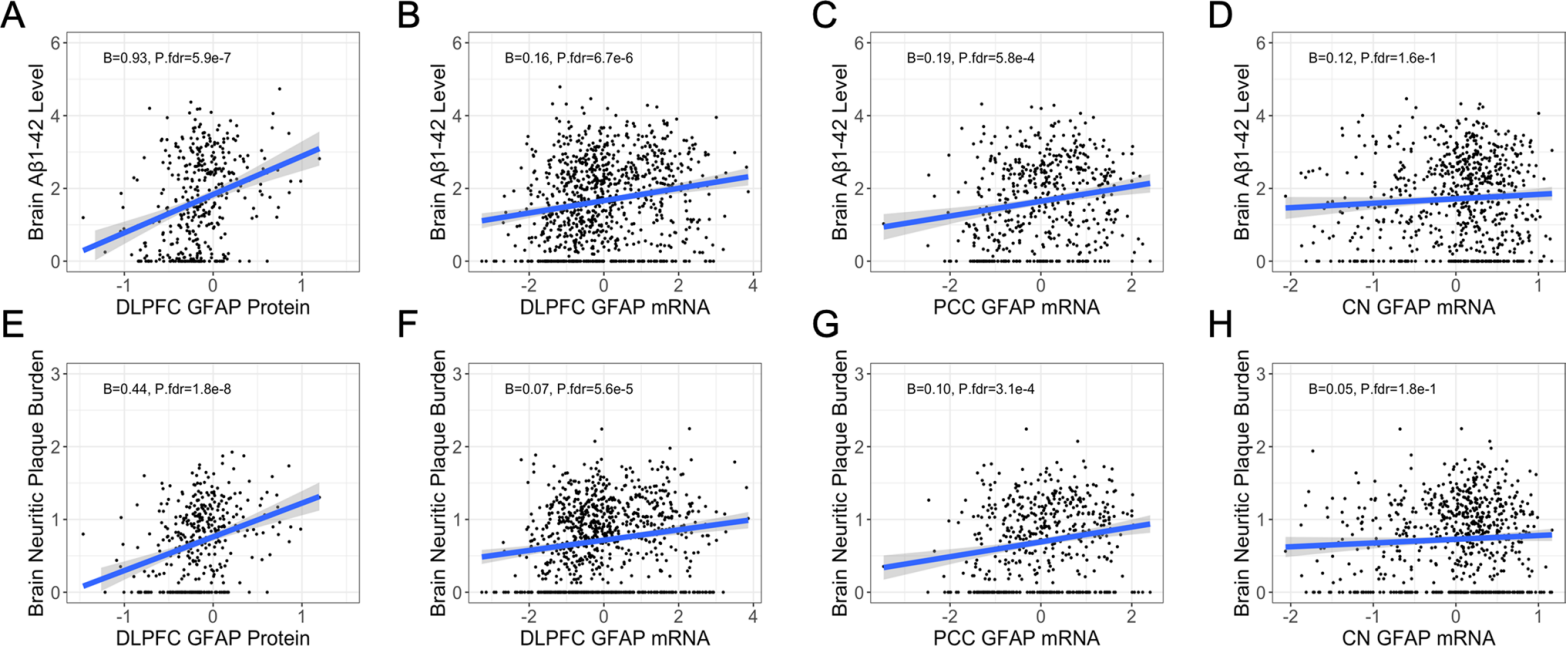
GFAP associations with brain amyloid. Cortical but not caudate GFAP is positively associated with brain amyloid burden. **A–D** Regional GFAP protein and mRNA levels by Aβ1-42 burden as measured by immunohistochemistry. **E–H** Regional GFAP protein and mRNA levels by neuritic plaque burden as measured by silver stain. Unadjusted scatter plots and statistical results from linear regression models adjusting for age at death, sex, and post-mortem interval

*GFAP* mRNA levels were associated with higher amyloid burden in both cortical regions and across both amyloid measures, with similar findings at the protein level (Table 2 and Fig. 2). Furthermore, the association with amyloid remained significant after adjusting for astrocytic transcript fraction in the dlPFC, providing evidence for strong upregulation of GFAP within astrocytes (Table 2). We obtained similar findings when assessing the effect of GFAP expression on tau pathology (Table 2). However, the association of *GFAP* mRNA expression and tau was attenuated after controlling for amyloid pathology, indicating that the effect of *GFAP* mRNA on tau is largely mediated by amyloid (Supplementary Table 1). Interestingly, the association remained significant in the PCC, potentially highlighting a unique relationship between *GFAP* mRNA and tau in this brain region (Supplementary Table 1). *GFAP* mRNA levels in the CN were not associated with any of the amyloid or tau outcome measures (Table 2).

**Table 2:** Main effects of GFAP expression on AD core pathology

Predictor	Outcome	*β*	SE	*p* value	P.fdr
dlPFC mRNA	Aβ_1-42_	0.157	0.0296	**1.34e−07**	**2.94e−06**
dlPFC mRNA	Neuritic plaque	0.0668	0.0140	**2.25e−06**	**3.71e−05**
dlPFC protein	Aβ_1-42_	0.934	0.170	**7.80e−8**	**2.57e−06**
dlPFC protein	Neuritic plaque	0.439	0.0705	**1.20e−9**	**7.92e−08**
CN mRNA	Aβ_1-42_	0.119	0.0702	9.08e−02	1.43e−01
CN mRNA	Neuritic plaque	0.0520	0.0322	1.07e−01	1.64e−01
PCC mRNA	Aβ_1-42_	0.191	0.0497	**1.39e−04**	**5.75e−04**
PCC mRNA	Neuritic plaque	0.0965	0.0234	**4.33e−05**	**2.86e−04**
dlPFC mRNA	p-Tau, AT8	0.138	0.0352	**9.23e−05**	**4.06e−04**
dlPFC mRNA	Neurofibrillary tangles	0.0437	0.0108	**5.90e−05**	**3.24e−04**
dlPFC protein	p-Tau, AT8	0.479	0.142	**7.99e−04**	**2.64e−03**
dlPFC protein	Neurofibrillary tangles	0.213	0.0522	**5.45e−05**	**3.24e−04**
CN mRNA	p-Tau, AT8	0.104	0.0797	1.92e−01	2.76e−01
CN mRNA	Neurofibrillary tangles	0.0331	0.0249	1.84e−01	2.76e−01
PCC mRNA	p-Tau, AT8	0.217	0.0548	**8.78e−05**	**4.06e−04**
PCC mRNA	Neurofibrillary tangles	0.0723	0.0173	**3.39e−05**	**2.68e−04**
Models adjusting for astrocyte transcript fraction					
dlPFC mRNA	Aβ_1-42_	0.2178	0.0464	**3.57e−06**	**4.71e−05**
dlPFC mRNA	Neuritic plaque	0.1004	0.0224	**9.79e−06**	**9.86e−05**
dlPFC mRNA	p-Tau, AT8	0.184	0.0532	**5.91e−04**	**2.15e−03**
dlPFC mRNA	Neurofibrillary tangles	0.0587	0.0164	**3.72e−04**	**1.44e−03**

Boldface signifies *p* < 0.05

*dlPFC* dorsolateral prefrontal cortex, *CN* caudate nucleus, *PCC* posterior cingulate cortex

### Cortical GFAP expression relates to a faster rate of global cognitive decline

**Fig. 3:**
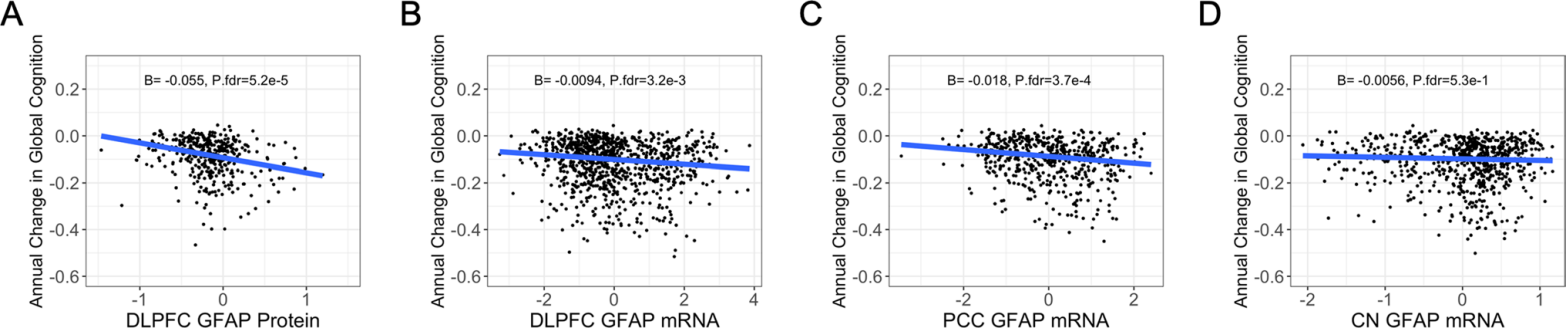
GFAP associations with cognitive decline. **A–D** High cortical but not caudate GFAP is associated with a faster rate of global cognitive decline. Unadjusted scatter plots and statistical results from linear mixed-effects regression models adjusting for age at death, sex, education, time from last study visit to death, and post-mortem interval. Time was represented as years from the final visit, with both time and intercept included as fixed and random effects

Next, we sought to determine if GFAP mRNA and protein expression were associated with global cognition cross-sectionally and longitudinally. Indeed, cortical GFAP was associated with worse global cognition at the final visit before death and a faster rate of cognitive decline in the years preceding death (Table 3; Fig. 3). These associations remained strong after controlling for astrocytic transcript fraction but did not survive p-value correction (Table 3). When controlling for pathology, the association between cortical GFAP protein levels and longitudinal cognition remained significant (Supplementary Table 1).

**Table 3:** Main effects of GFAP expression on cognition

Predictor	Outcome	*β*	SE	*p* value	P.fdr
dlPFC mRNA	Cross-sectional cognition	−0.0599	0.0301	**4.67e−02**	9.06e−02
dlPFC mRNA	Longitudinal cognition	−0.0094	0.0029	**1.10e−03**	**3.29e−03**
dlPFC protein	Cross-sectional cognition	−0.423	0.155	**6.83e−03**	**1.88e−02**
dlPFC protein	Longitudinal cognition	−0.0549	0.0124	**1.046e−05**	**9.86e−05**
CN mRNA	Cross-sectional cognition	−0.0593	0.0670	3.77e−01	4.88e−01
CN mRNA	Longitudinal cognition	−0.0056	0.0066	3.92e−01	4.91e−01
PCC mRNA	Cross-sectional cognition	−0.1584	0.0476	**9.54e−04**	**2.30e−03**
PCC mRNA	Longitudinal cognition	−0.0176	0.0044	**6.69e−05**	**3.40e−04**
Models adjusting for astrocyte transcript fraction					
dlPFC mRNA	Cross-sectional cognition	−0.102	0.0464	**2.79e−2**	6.14e−02
dlPFC mRNA	Longitudinal cognition	−0.0107	0.00510	**3.52e−2**	7.37e−02

Boldface signifies *p* < 0.05

*dlPFC* dorsolateral prefrontal cortex, *CN* caudate nucleus, *PCC* posterior cingulate cortex

### Cortical GFAP expression is associated with cerebrovascular, TDP-43, and Lewy body pathologies

Following our main analyses leveraging core AD pathology, we examined the relationship between regional GFAP expression and a diverse group of pathologies. Higher cortical mRNA and protein expression of GFAP was associated with greater burden of CAA pathology, while there was no association of caudate *GFAP* mRNA with CAA pathology (Supplementary Table 2 and Supplementary Fig. 3). In addition, we observed significant associations with dlPFC mRNA expression of *GFAP* with macro infarcts and dlPFC protein with TDP-43 and Lewy body pathologies, although the associations with macroinfarcts and Lewy body pathology did not survive multiple test correction (Supplementary Table 2). Finally, in amyloid-stratified models, we observed non-significant associations of GFAP and non-AD pathologies (Supplementary Tables 3 and 4).

### GFAP interacts with amyloid on tau, longitudinal cognition

**Fig. 4:**
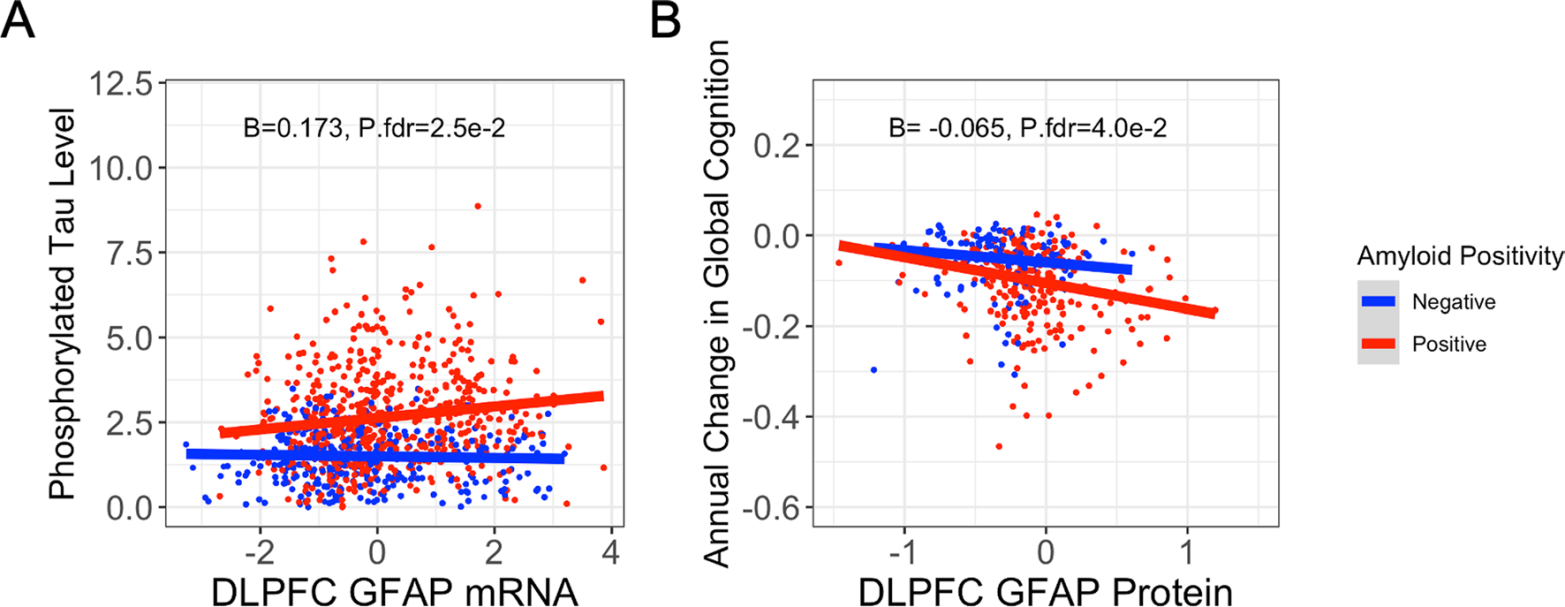
Significant interactions of GFAP expression and amyloid status. **A** High *GFAP* mRNA expression in the dlPFC relates to a high brain phosphorylated tau burden in amyloid-positive individuals. Phosphorylated tau was quantified by immunohistochemistry, and amyloid positivity was defined as CERAD “moderate” or “frequent.” Unadjusted scatter plots and statistical results from linear regression models adjusting for age at death, sex, and post-mortem interval. **B** High GFAP protein expression in the dlPFC relates to a faster rate of global cognitive decline in amyloid-positive individuals. Amyloid-positivity was defined as CERAD “moderate” or “frequent.” Unadjusted scatter plots and statistical results from linear mixed-effects regression models adjusting for age at death, sex, education, time from last study visit to death, and post-mortem interval. Time was represented as years from the final visit, with both time and intercept included as fixed and random effects

Finally, we sought to characterize the interaction between brain GFAP expression and amyloid on components of AD downstream of amyloid deposition, including tau pathology and cognitive decline. Indeed, we observed a significant interaction between amyloid and dlPFC *GFAP* mRNA on tau, such that high *GFAP* expression related to high phosphorylated tau burden only in amyloid-positive individuals (Table 4; Fig. 4). Similarly, when testing if amyloid and GFAP levels interacted on longitudinal cognition, we observed a significant interaction in the dlPFC between GFAP protein levels and amyloid status, such that high GFAP related to a faster rate of cognitive decline in amyloid-positive individuals (Table 4; Fig. 4).

**Table 4:** Interactions of GFAP and amyloid status on tau and cognition

Predictor	Outcome	*β*	SE	*p* value	P.fdr
dlPFC mRNA x amyloid status	p-Tau, AT8	0.173	0.0665	**9.36e−03**	**2.47e−02**
dlPFC protein x amyloid status	p-Tau, AT8	0.506	0.295	8.70e−02	1.40e−01
CN mRNA x amyloid status	p-Tau, AT8	0.0716	0.149	6.31e−01	7.31e−01
PCC mRNA x amyloid status	p-Tau, AT8	0.196	0.103	5.60e−02	1.03e−01
dlPFC mRNA x amyloid status	Longitudinal cognition	−0.0118	0.0056	**3.58e−02**	7.37e−02
dlPFC protein x amyloid status	Longitudinal cognition	−0.0649	0.0272	**1.71e−02**	**4.03e−02**
CN mRNA x amyloid status	Longitudinal cognition	0.0003	0.0128	9.82e−01	9.93e−01
PCC mRNA x amyloid status	Longitudinal cognition	−0.0035	0.0086	6.86e−01	7.67e−01

Boldface signifies *p* < 0.05

Amyloid status is defined by a binary variable where amyloid negativity = CERAD “none” or “sparse” and amyloid positivity = CERAD “moderate” or frequent”

*dlPFC* dorsolateral prefrontal cortex, *CN* caudate nucleus, *PCC* posterior cingulate cortex

## Discussion

Our study provides comprehensive insights into the role of brain region-specific GFAP expression in Alzheimer’s disease using bulk mRNA sequencing and TMT-MS proteomics. We observed significant associations between cortical GFAP expression and multiple clinical and pathological features of AD. Specifically, we found that GFAP expression in the dorsolateral prefrontal cortex and posterior cingulate cortex was significantly elevated in individuals with clinical and pathologically confirmed AD, while such differences were not observed in the caudate nucleus. The lack of GFAP upregulation in the caudate may be due to the fact that this region exhibits amyloid deposition later in the disease stage than cortical regions, thus reflecting a lower level of astrocyte reactivity [47]. Interestingly, the density of GFAP-immunoreactive astrocytes in non-demented elderly adults has previously been shown to be higher in the caudate than cortical regions, potentially influencing the coordinated astrocyte response to insult [29]. Cortical GFAP expression was strongly associated with amyloid pathology, tau pathology, and a faster rate of cognitive decline. Furthermore, GFAP associations with phosphorylated tau burden and cognition were modified by amyloid burden, such that the association was most pronounced among amyloid-positive individuals, confirming the previous observations leveraging *in vivo* biomarkers. These findings highlight patterns of astrocyte reactivity that may respond to the spread of amyloid pathology in the brain, signaling changes that precede the onset of AD diagnosis.

While numerous studies have assessed the viability of elevated plasma GFAP as a biomarker for AD and amyloid pathology [1, 3, 11, 14, 17, 37, 43, 49], few have focused on changes in GFAP expression in brain. Here, we report increased GFAP levels in the dlPFC and PCC of AD patients compared to those with no cognitive impairment, reinforcing the utility of GFAP as a blood-based biomarker in AD and confirming that measures collected from the periphery reflect processes happening in the CNS. Notably, the finding that GFAP levels in the blood are most strongly related to cognitive decline among amyloid-positive individuals [3, 4, 33, 48] appears to be strongly supported by our data in the brain.

Across cortical regions, we found the evidence of strong associations between GFAP expression and amyloid pathology. This relationship remained statistically significant even after adjusting for astrocytic transcript fraction, underscoring the robust upregulation of GFAP within astrocytes in the presence of amyloid pathology. GFAP was also strongly associated with tau pathology, but the attenuated association between *GFAP* mRNA and tau burden after controlling for amyloid pathology suggests that GFAP’s effect on tau may be mediated through amyloid pathways. Furthermore, we observed a significant interaction between *GFAP* mRNA in the dlPFC and amyloid status on tau burden, indicating that amyloid status modifies the effect of GFAP expression on tau. As such, amyloid-positive individuals with high GFAP expression displayed a greater level of tau burden than amyloid-negative individuals, supporting the notion that astrocyte reactivity may serve as an intermediary between amyloid and tau pathology [3]. While studies in rodent models of AD have shown mixed outcomes in response to attenuation of astrocyte reactivity, chronic activation states of astrocytes in humans may ultimately be detrimental and exacerbate pathology and inflammatory signaling [10, 19, 23, 32, 35]. This highlights the importance of astrocytic involvement in the amyloid cascade hypothesis and warrants the further investigation of attenuation of astrocyte reactivity as a therapeutic target in AD.

Interestingly, we observed lower GFAP expression in Aβ-/tau+ individuals compared to Aβ+/tau+ individuals, suggesting that GFAP is more closely linked to amyloid than tau pathology. This is supported by the fact that the association of GFAP expression and tau was attenuated when covarying for amyloid pathology. As such, in the context of the amyloid cascade hypothesis, GFAP upregulation may occur early in disease progression, reinforcing the utility of plasma GFAP as a biomarker sensitive to pathologic changes in brain. Furthermore, increased GFAP expression does not appear to be linked to tau positivity in the absence of amyloid pathology, potentially reflecting astrocytic responses to extracellular amyloid plaques vs intracellular neurofibrillary tangles. This upregulation was most pronounced in Aβ+/tau+ individuals, reflecting the high degree of amyloid burden present in these individuals and possibly indicating a combinatorial effect between amyloid and tau pathology.

We observed that higher cortical GFAP expression correlates with worse global cognition at the visit prior to death and a faster rate of cognitive decline. The significant interaction between GFAP protein levels and amyloid status on longitudinal cognition further supports the role of GFAP in modulating the cognitive effects of amyloid pathology. Accordingly, a high degree of astrocyte reactivity in amyloid-positive individuals relates to a faster rate of global cognitive decline. Identifying genetic factors that influence the degree of astrocyte reactivity may inform precision-medicine approaches and enable early, targeted interventions.

In addition to the core AD pathologies, we identified significant associations of GFAP expression with other neuropathologies, including cerebrovascular disease, TDP-43, and Lewy body pathologies. Most prominently, elevated GFAP levels correlated with greater burden of cerebral amyloid angiopathy (CAA), which is intuitive given the strong associations of GFAP expression and global amyloid. In amyloid-stratified models, we observed attenuated associations with all non-AD pathologies, potentially due to a reduction in statistical power. However, the directions-of-effect with CAA pathology in amyloid-positive participants were largely consistent with our main analyses, further indicating that the association between GFAP and CAA stage depends on the presence of amyloid pathology. High GFAP is also related to the presence of TDP-43 pathology extending beyond the amygdala. Finally, high GFAP was correlated with the presence of macroinfarcts and neocortical Lewy body pathology, although these associations did not survive correction for multiple comparisons. The connection between cerebral infarcts and astrocyte reactivity is well established, and serum GFAP levels have been shown to correlate with severity of acute ischemic stroke and poorer clinical outcomes [34, 51]. Conversely, the astrocytic response to TDP-43 is not as thoroughly characterized, although past work has shown that astrocytes with TDP-43 inclusions resist conversion to a reactive state early in disease progression in *in vitro* models of amyotrophic lateral sclerosis [44]. Another study found no relationship between antemortem plasma GFAP and post-mortem TDP-43 pathology [36]. Finally, plasma GFAP has been shown to be higher in autopsy-confirmed α-synuclein positive Lewy body spectrum disorders with concomitant AD pathology versus AD pathology alone [12]. Our findings align with the previous literature suggesting astrocytic involvement in various neuropathological processes, indicating that targeting astrocyte reactivity may have therapeutic implications beyond canonical AD [20, 22, 25, 42]. Ultimately, investigating the temporal nature of astrocyte responses to various insults will be critical for determining whether such responses are beneficial or harmful, as comorbid pathologies can both amplify the extent and lengthen the timeline of astrocyte reactivity.

The present analyses have numerous strengths. ROS/MAP is a well-characterized and deeply phenotyped longitudinal study, with our included sample representing an average follow-up time of 7.5–9 years. We included analyses of numerous non-AD pathologies, providing more insight into conditions that often co-occur in pathological aging. Furthermore, we provided multiple levels of validation for the observed associations by leveraging both mRNA and protein-based measures of GFAP expression from multiple brain regions. Despite these strengths, our study features some limitations. These include the cross-sectional nature of the pathologic, transcriptomic, and proteomic data, making it difficult to discern causality. In addition, measurement of mRNA transcript levels does not necessarily translate to protein expression. While it is encouraging that we observed largely consistent findings in the dlPFC, which featured both mRNA and protein measurements in overlapping samples, the correlation between mRNA transcripts and protein was 0.25. This indicates that a sizable portion of transcripts may not translate to functional protein. In addition, examining auxiliary neocortical regions beyond the DLPFC may yield unique insights to domain-specific cognitive function (i.e., temporal cortex and episodic memory), as we leveraged a global cognition composite rather than unique domains. Expansion of our analyses to additional cohort studies which feature different brain regions than the present study remains a priority for future work. While GFAP did show expected correlations with certain cytokines/chemokines known to be released by reactive astrocytes, future work validating GFAP protein alterations in blood and brain in those with and without noted astrocyte activation and their association with inflammatory protein levels will be needed to confirm our interpretations. A further limitation is that participants were predominantly of European ancestry, which may limit the generalizability of our findings. Finally, it is possible we are missing some region-specific effects due to the lack of amyloid and tau measurements in the PCC and CN. Future studies should aim to include more diverse populations and assess GFAP associations with pathology cis-regionally to better understand the spatial and temporal dynamics of GFAP expression in relation to AD progression.

In sum, our study demonstrates that region-specific GFAP expression in the brain significantly correlates with various clinical and pathological features of Alzheimer’s disease, particularly in the cortex. These findings support the use of GFAP as a potential biomarker for AD and emphasize the role of astrocyte reactivity in the disease’s pathology. Future studies exploring the role of genetic factors in astrocyte activation and how astrocyte reactivity contributes to the development of tau pathology will be crucial in moving toward targeted, precision interventions.

## Supplementary Information

Below is the link to the electronic supplementary material.

Supplementary file1 (PPTX 3409 KB)

## Data Availability

ROSMAP data are available at www.radc.rush.edu.
