# Supplementary material for: Pathologic and clinical correlates of region-specific brain GFAP in Alzheimer’s disease
Source: Acta Neuropathol. 2024 Nov 24;148(1):69. doi: 10.1007/s00401-024-02828-5 (PMC11586308; doi:10.1007/s00401-024-02828-5)
Supplement: Supplementary file 1 — Supplementary file1 (PPTX 3409 KB) [file 401_2024_2828_MOESM1_ESM.pptx]

## Slide 1
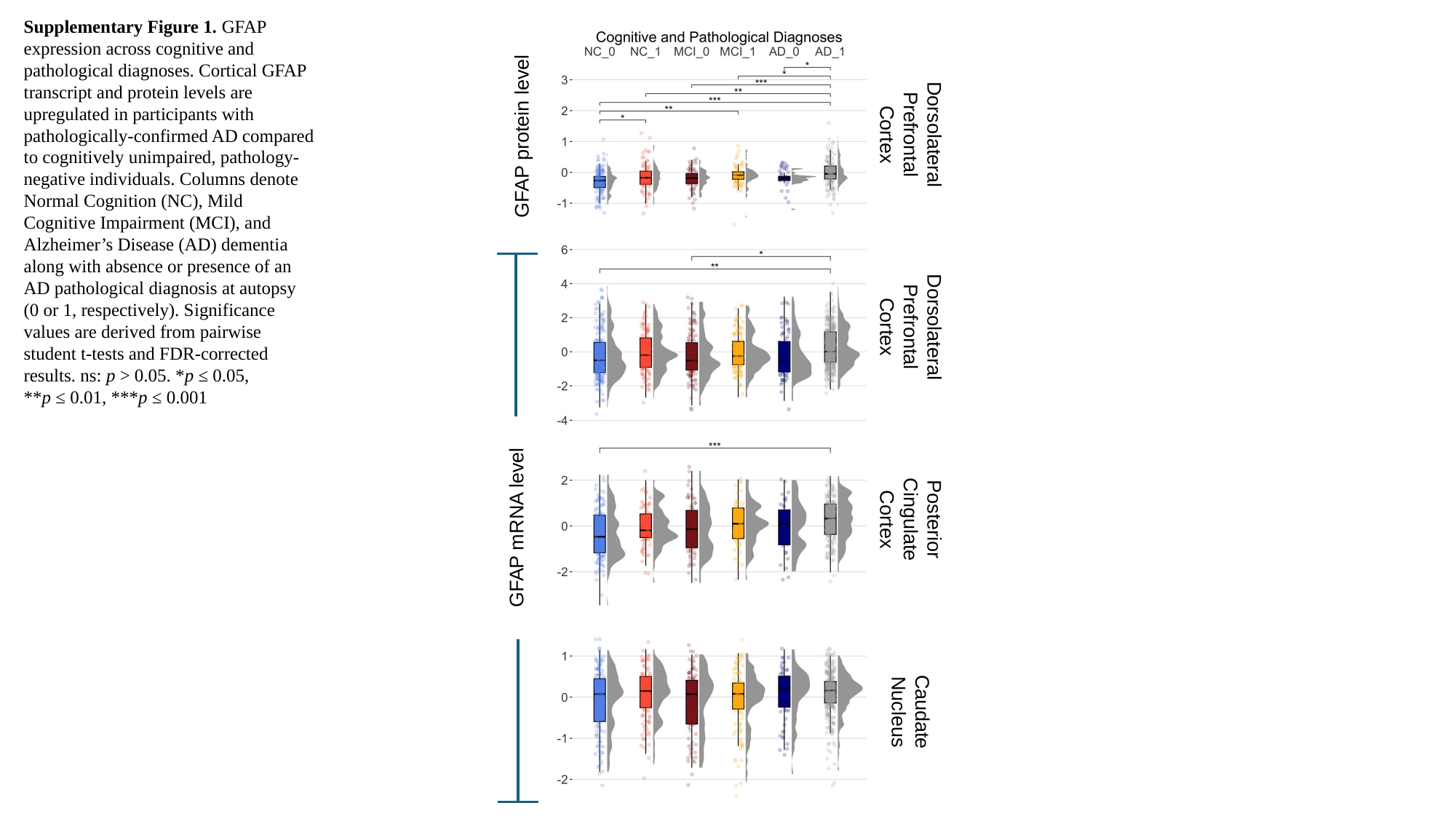

Supplementary Figure 1. GFAP expression across cognitive and pathological diagnoses. Cortical GFAP transcript and protein levels are upregulated in participants with pathologically-confirmed AD compared to cognitively unimpaired, pathology-negative individuals. Columns denote Normal Cognition (NC), Mild Cognitive Impairment (MCI), and Alzheimer’s Disease (AD) dementia along with absence or presence of an AD pathological diagnosis at autopsy (0 or 1, respectively). Significance values are derived from pairwise student t-tests and FDR-corrected results. ns: p > 0.05. *p ≤ 0.05, **p ≤ 0.01, ***p ≤ 0.001
Dorsolateral
Prefrontal
Cortex
GFAP protein level
Dorsolateral
Prefrontal
Cortex
Posterior
Cingulate
Cortex
GFAP mRNA level
Caudate Nucleus

## Slide 2
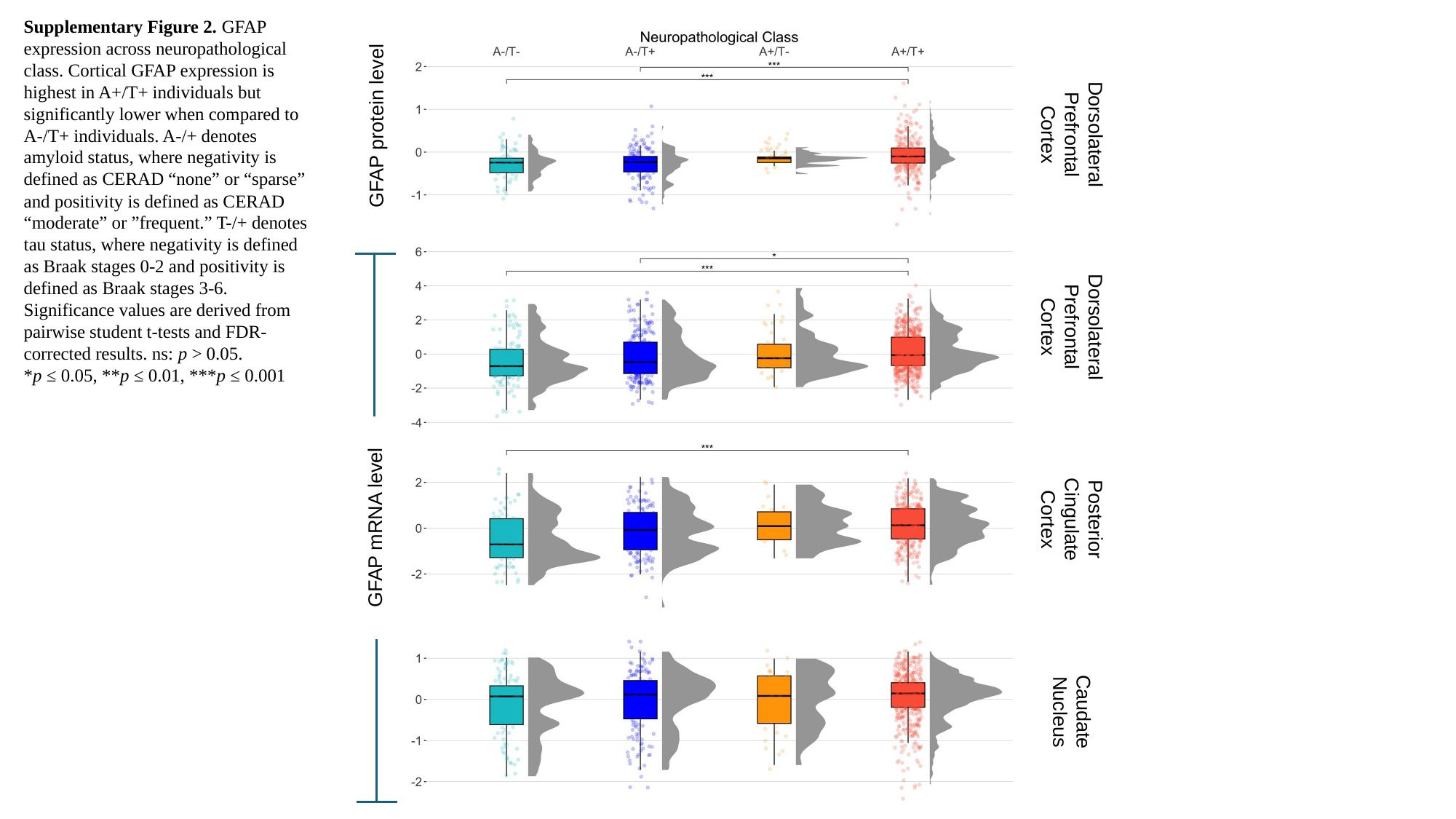

Supplementary Figure 2. GFAP expression across neuropathological class. Cortical GFAP expression is highest in A+/T+ individuals but significantly lower when compared to A-/T+ individuals. A-/+ denotes amyloid status, where negativity is defined as CERAD “none” or “sparse” and positivity is defined as CERAD “moderate” or ”frequent.” T-/+ denotes tau status, where negativity is defined as Braak stages 0-2 and positivity is defined as Braak stages 3-6. Significance values are derived from pairwise student t-tests and FDR-corrected results. ns: p > 0.05. *p ≤ 0.05, **p ≤ 0.01, ***p ≤ 0.001
Dorsolateral
Prefrontal
Cortex
GFAP protein level
Dorsolateral
Prefrontal
Cortex
Posterior
Cingulate
Cortex
GFAP mRNA level
Caudate Nucleus

## Slide 3
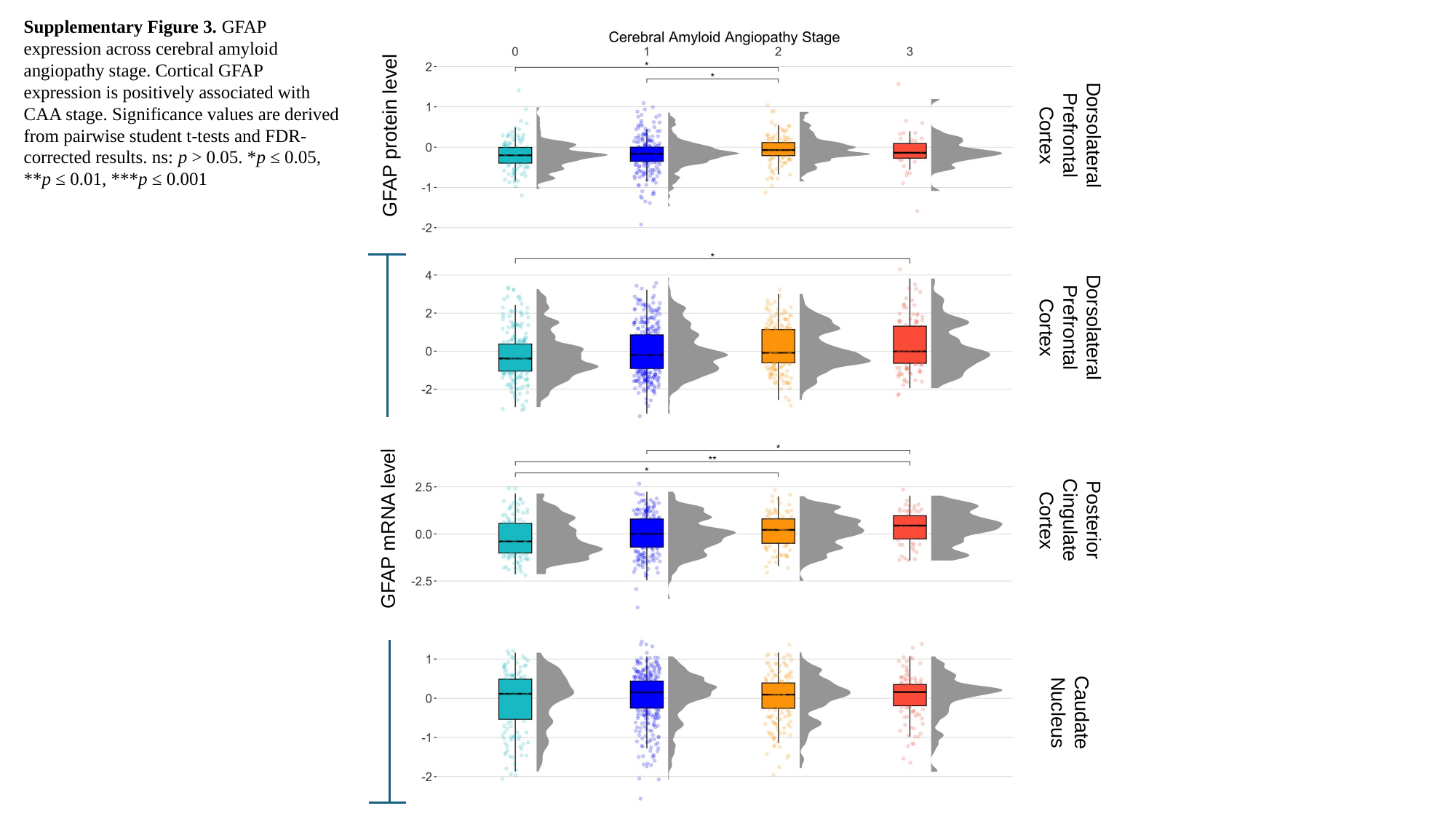

Supplementary Figure 3. GFAP expression across cerebral amyloid angiopathy stage. Cortical GFAP expression is positively associated with CAA stage. Significance values are derived from pairwise student t-tests and FDR-corrected results. ns: p > 0.05. *p ≤ 0.05, **p ≤ 0.01, ***p ≤ 0.001
Dorsolateral
Prefrontal
Cortex
GFAP protein level
Dorsolateral
Prefrontal
Cortex
Posterior
Cingulate
Cortex
GFAP mRNA level
Caudate Nucleus

## Slide 4
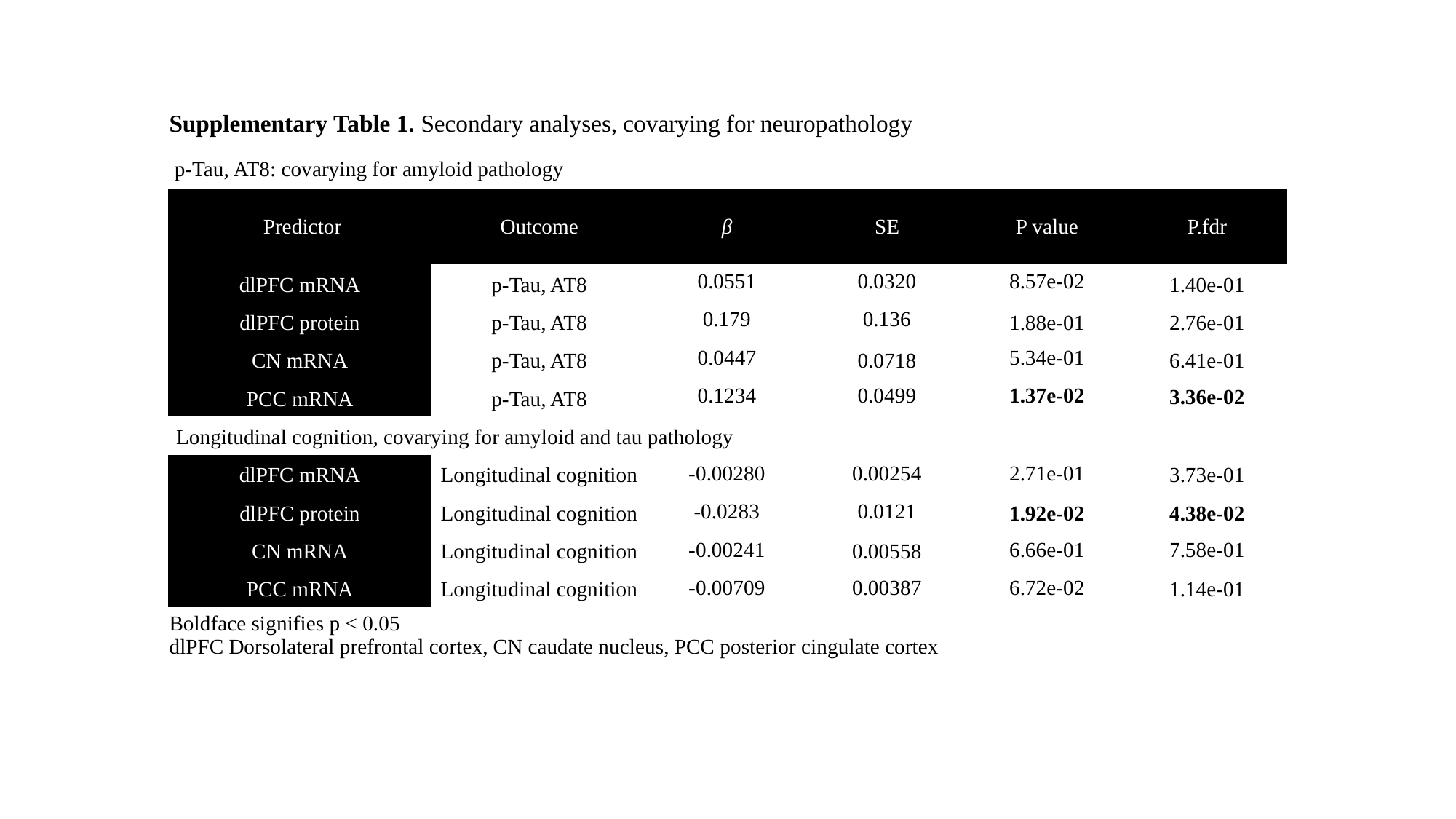

| Supplementary Table 1. Secondary analyses, covarying for neuropathology | | | | | |
| --- | --- | --- | --- | --- | --- |
| p-Tau, AT8: covarying for amyloid pathology | | | | | |
| Predictor | Outcome | β | SE | P value | P.fdr |
| dlPFC mRNA | p-Tau, AT8 | 0.0551 | 0.0320 | 8.57e-02 | 1.40e-01 |
| dlPFC protein | p-Tau, AT8 | 0.179 | 0.136 | 1.88e-01 | 2.76e-01 |
| CN mRNA | p-Tau, AT8 | 0.0447 | 0.0718 | 5.34e-01 | 6.41e-01 |
| PCC mRNA | p-Tau, AT8 | 0.1234 | 0.0499 | 1.37e-02 | 3.36e-02 |
| Longitudinal cognition, covarying for amyloid and tau pathology | | | | | |
| dlPFC mRNA | Longitudinal cognition | -0.00280 | 0.00254 | 2.71e-01 | 3.73e-01 |
| dlPFC protein | Longitudinal cognition | -0.0283 | 0.0121 | 1.92e-02 | 4.38e-02 |
| CN mRNA | Longitudinal cognition | -0.00241 | 0.00558 | 6.66e-01 | 7.58e-01 |
| PCC mRNA | Longitudinal cognition | -0.00709 | 0.00387 | 6.72e-02 | 1.14e-01 |
| Boldface signifies p < 0.05 dlPFC Dorsolateral prefrontal cortex, CN caudate nucleus, PCC posterior cingulate cortex | | | | | |

## Slide 5
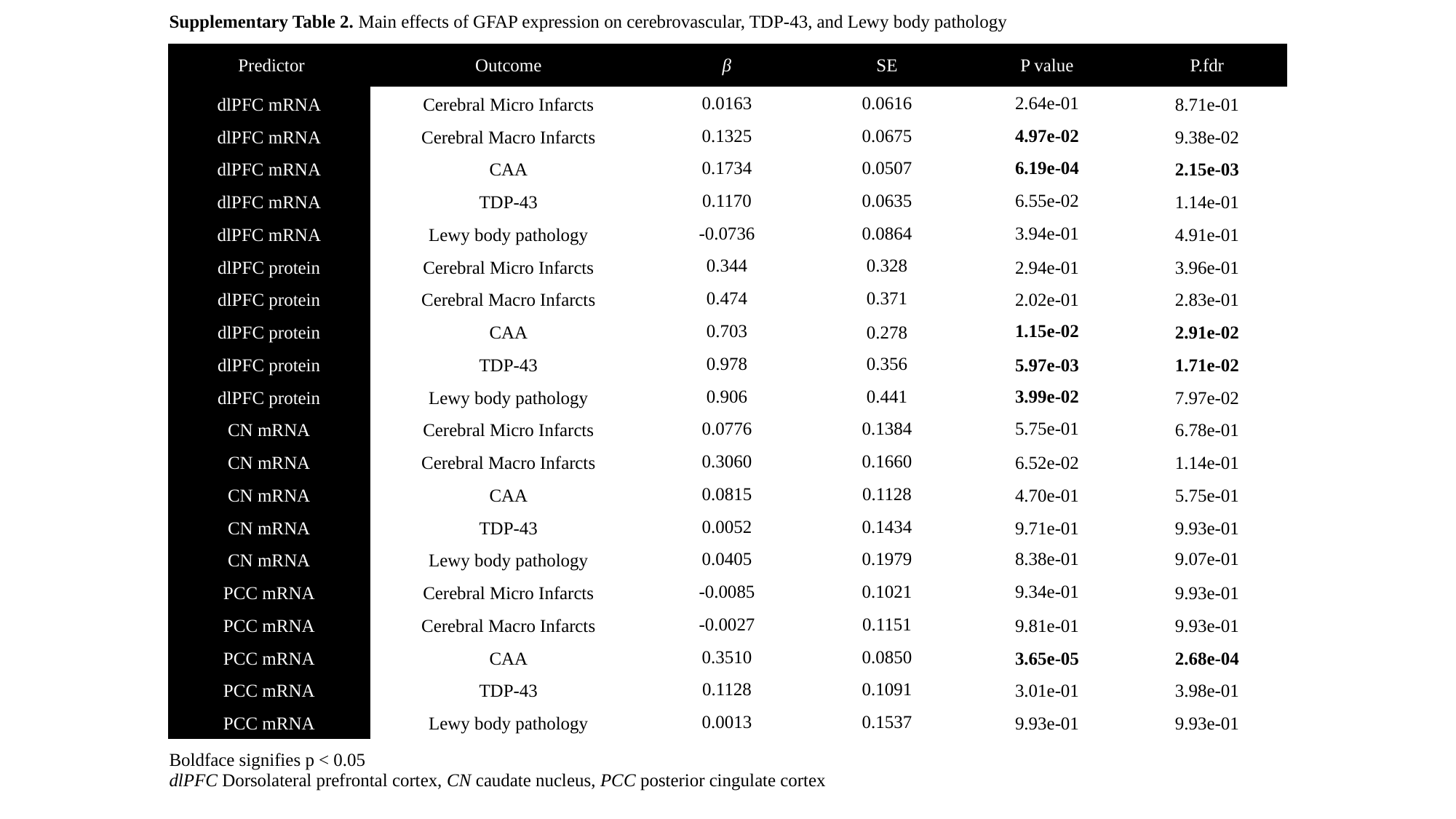

| Supplementary Table 2. Main effects of GFAP expression on cerebrovascular, TDP-43, and Lewy body pathology | | | | | |
| --- | --- | --- | --- | --- | --- |
| Predictor | Outcome | β | SE | P value | P.fdr |
| dlPFC mRNA | Cerebral Micro Infarcts | 0.0163 | 0.0616 | 2.64e-01 | 8.71e-01 |
| dlPFC mRNA | Cerebral Macro Infarcts | 0.1325 | 0.0675 | 4.97e-02 | 9.38e-02 |
| dlPFC mRNA | CAA | 0.1734 | 0.0507 | 6.19e-04 | 2.15e-03 |
| dlPFC mRNA | TDP-43 | 0.1170 | 0.0635 | 6.55e-02 | 1.14e-01 |
| dlPFC mRNA | Lewy body pathology | -0.0736 | 0.0864 | 3.94e-01 | 4.91e-01 |
| dlPFC protein | Cerebral Micro Infarcts | 0.344 | 0.328 | 2.94e-01 | 3.96e-01 |
| dlPFC protein | Cerebral Macro Infarcts | 0.474 | 0.371 | 2.02e-01 | 2.83e-01 |
| dlPFC protein | CAA | 0.703 | 0.278 | 1.15e-02 | 2.91e-02 |
| dlPFC protein | TDP-43 | 0.978 | 0.356 | 5.97e-03 | 1.71e-02 |
| dlPFC protein | Lewy body pathology | 0.906 | 0.441 | 3.99e-02 | 7.97e-02 |
| CN mRNA | Cerebral Micro Infarcts | 0.0776 | 0.1384 | 5.75e-01 | 6.78e-01 |
| CN mRNA | Cerebral Macro Infarcts | 0.3060 | 0.1660 | 6.52e-02 | 1.14e-01 |
| CN mRNA | CAA | 0.0815 | 0.1128 | 4.70e-01 | 5.75e-01 |
| CN mRNA | TDP-43 | 0.0052 | 0.1434 | 9.71e-01 | 9.93e-01 |
| CN mRNA | Lewy body pathology | 0.0405 | 0.1979 | 8.38e-01 | 9.07e-01 |
| PCC mRNA | Cerebral Micro Infarcts | -0.0085 | 0.1021 | 9.34e-01 | 9.93e-01 |
| PCC mRNA | Cerebral Macro Infarcts | -0.0027 | 0.1151 | 9.81e-01 | 9.93e-01 |
| PCC mRNA | CAA | 0.3510 | 0.0850 | 3.65e-05 | 2.68e-04 |
| PCC mRNA | TDP-43 | 0.1128 | 0.1091 | 3.01e-01 | 3.98e-01 |
| PCC mRNA | Lewy body pathology | 0.0013 | 0.1537 | 9.93e-01 | 9.93e-01 |
| Boldface signifies p < 0.05 dlPFC Dorsolateral prefrontal cortex, CN caudate nucleus, PCC posterior cingulate cortex | | | | | |

## Slide 6
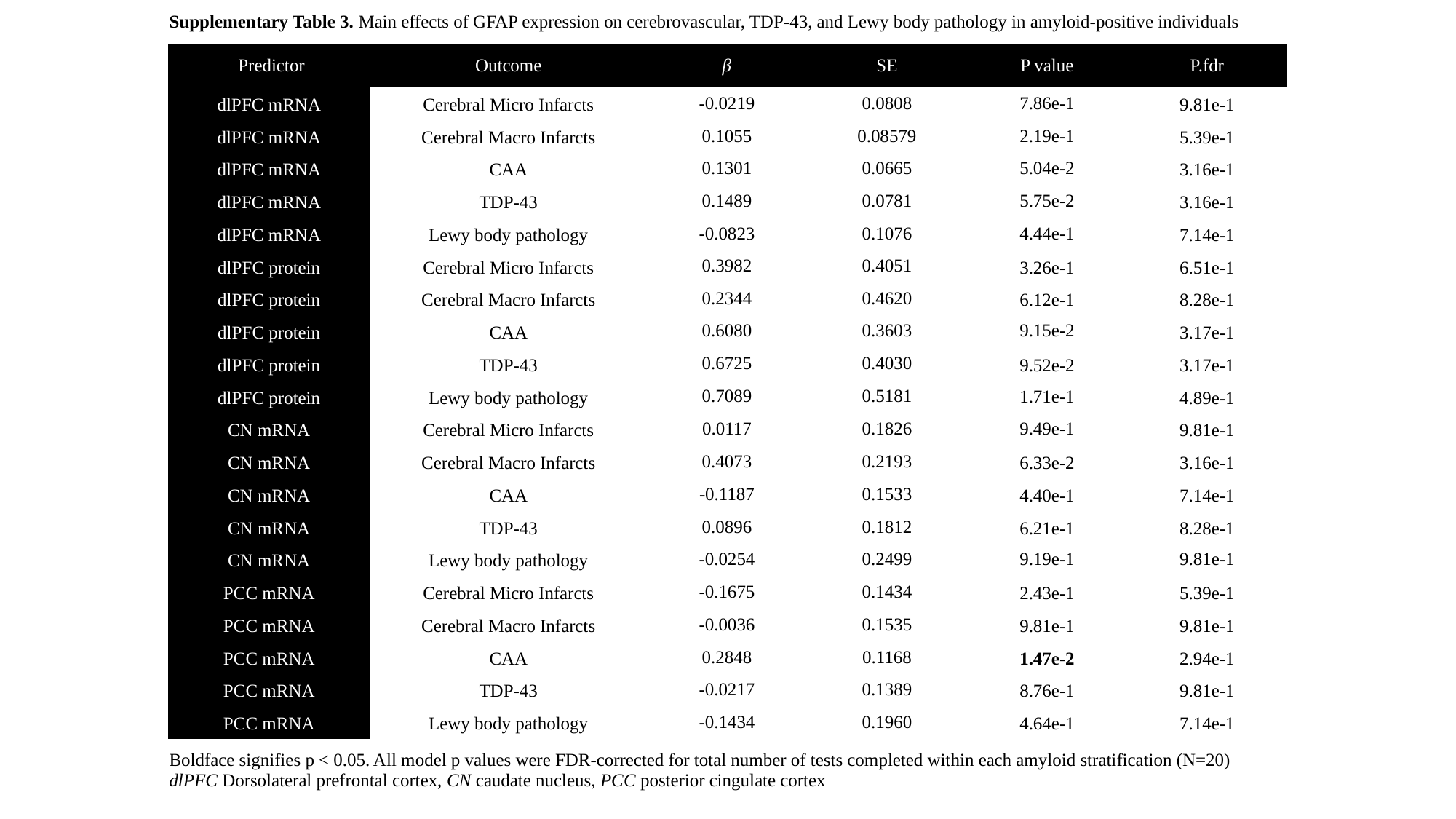

| Supplementary Table 3. Main effects of GFAP expression on cerebrovascular, TDP-43, and Lewy body pathology in amyloid-positive individuals | | | | | |
| --- | --- | --- | --- | --- | --- |
| Predictor | Outcome | β | SE | P value | P.fdr |
| dlPFC mRNA | Cerebral Micro Infarcts | -0.0219 | 0.0808 | 7.86e-1 | 9.81e-1 |
| dlPFC mRNA | Cerebral Macro Infarcts | 0.1055 | 0.08579 | 2.19e-1 | 5.39e-1 |
| dlPFC mRNA | CAA | 0.1301 | 0.0665 | 5.04e-2 | 3.16e-1 |
| dlPFC mRNA | TDP-43 | 0.1489 | 0.0781 | 5.75e-2 | 3.16e-1 |
| dlPFC mRNA | Lewy body pathology | -0.0823 | 0.1076 | 4.44e-1 | 7.14e-1 |
| dlPFC protein | Cerebral Micro Infarcts | 0.3982 | 0.4051 | 3.26e-1 | 6.51e-1 |
| dlPFC protein | Cerebral Macro Infarcts | 0.2344 | 0.4620 | 6.12e-1 | 8.28e-1 |
| dlPFC protein | CAA | 0.6080 | 0.3603 | 9.15e-2 | 3.17e-1 |
| dlPFC protein | TDP-43 | 0.6725 | 0.4030 | 9.52e-2 | 3.17e-1 |
| dlPFC protein | Lewy body pathology | 0.7089 | 0.5181 | 1.71e-1 | 4.89e-1 |
| CN mRNA | Cerebral Micro Infarcts | 0.0117 | 0.1826 | 9.49e-1 | 9.81e-1 |
| CN mRNA | Cerebral Macro Infarcts | 0.4073 | 0.2193 | 6.33e-2 | 3.16e-1 |
| CN mRNA | CAA | -0.1187 | 0.1533 | 4.40e-1 | 7.14e-1 |
| CN mRNA | TDP-43 | 0.0896 | 0.1812 | 6.21e-1 | 8.28e-1 |
| CN mRNA | Lewy body pathology | -0.0254 | 0.2499 | 9.19e-1 | 9.81e-1 |
| PCC mRNA | Cerebral Micro Infarcts | -0.1675 | 0.1434 | 2.43e-1 | 5.39e-1 |
| PCC mRNA | Cerebral Macro Infarcts | -0.0036 | 0.1535 | 9.81e-1 | 9.81e-1 |
| PCC mRNA | CAA | 0.2848 | 0.1168 | 1.47e-2 | 2.94e-1 |
| PCC mRNA | TDP-43 | -0.0217 | 0.1389 | 8.76e-1 | 9.81e-1 |
| PCC mRNA | Lewy body pathology | -0.1434 | 0.1960 | 4.64e-1 | 7.14e-1 |
| Boldface signifies p < 0.05. All model p values were FDR-corrected for total number of tests completed within each amyloid stratification (N=20) dlPFC Dorsolateral prefrontal cortex, CN caudate nucleus, PCC posterior cingulate cortex | | | | | |

## Slide 7
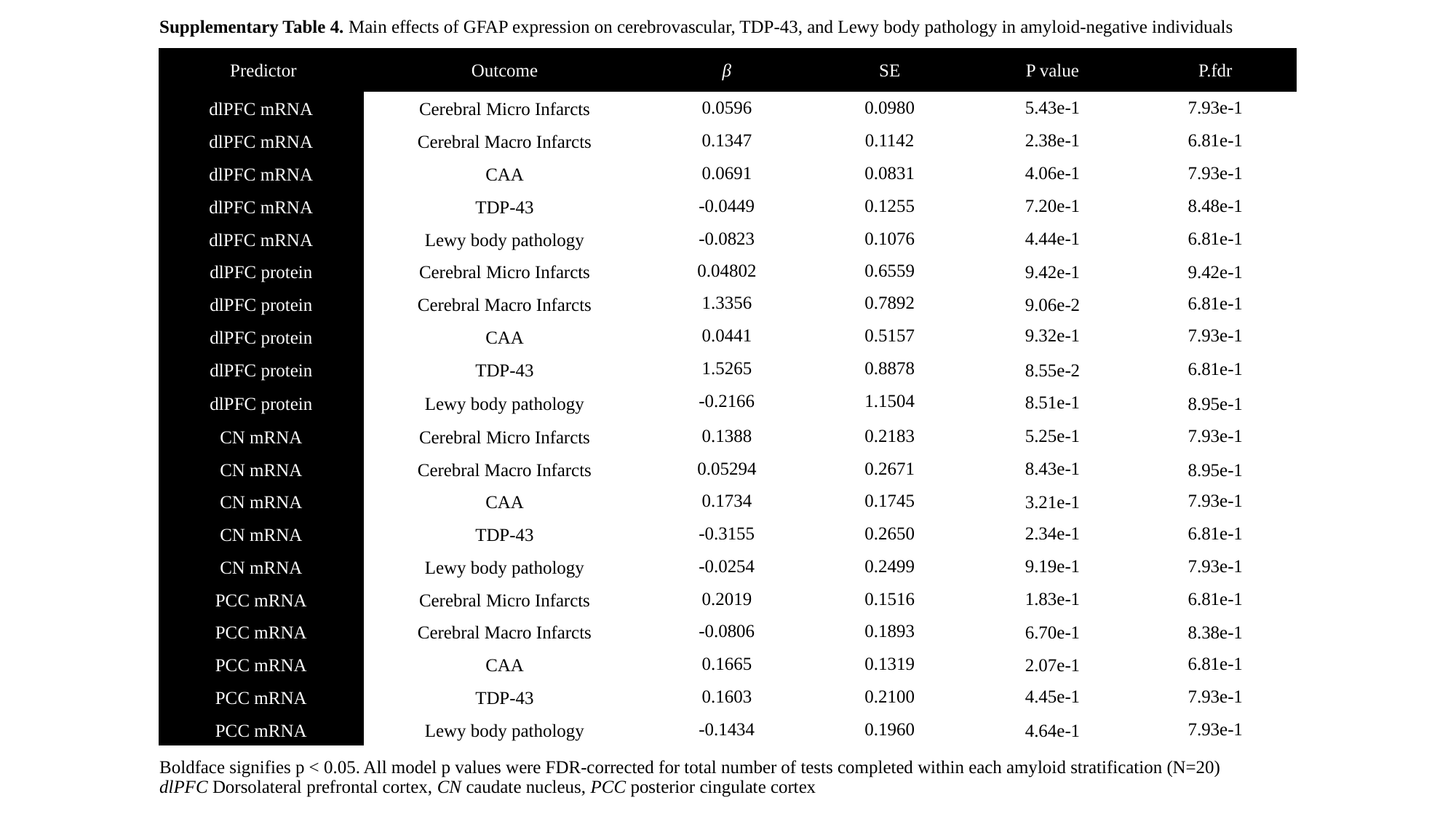

| Supplementary Table 4. Main effects of GFAP expression on cerebrovascular, TDP-43, and Lewy body pathology in amyloid-negative individuals | | | | | |
| --- | --- | --- | --- | --- | --- |
| Predictor | Outcome | β | SE | P value | P.fdr |
| dlPFC mRNA | Cerebral Micro Infarcts | 0.0596 | 0.0980 | 5.43e-1 | 7.93e-1 |
| dlPFC mRNA | Cerebral Macro Infarcts | 0.1347 | 0.1142 | 2.38e-1 | 6.81e-1 |
| dlPFC mRNA | CAA | 0.0691 | 0.0831 | 4.06e-1 | 7.93e-1 |
| dlPFC mRNA | TDP-43 | -0.0449 | 0.1255 | 7.20e-1 | 8.48e-1 |
| dlPFC mRNA | Lewy body pathology | -0.0823 | 0.1076 | 4.44e-1 | 6.81e-1 |
| dlPFC protein | Cerebral Micro Infarcts | 0.04802 | 0.6559 | 9.42e-1 | 9.42e-1 |
| dlPFC protein | Cerebral Macro Infarcts | 1.3356 | 0.7892 | 9.06e-2 | 6.81e-1 |
| dlPFC protein | CAA | 0.0441 | 0.5157 | 9.32e-1 | 7.93e-1 |
| dlPFC protein | TDP-43 | 1.5265 | 0.8878 | 8.55e-2 | 6.81e-1 |
| dlPFC protein | Lewy body pathology | -0.2166 | 1.1504 | 8.51e-1 | 8.95e-1 |
| CN mRNA | Cerebral Micro Infarcts | 0.1388 | 0.2183 | 5.25e-1 | 7.93e-1 |
| CN mRNA | Cerebral Macro Infarcts | 0.05294 | 0.2671 | 8.43e-1 | 8.95e-1 |
| CN mRNA | CAA | 0.1734 | 0.1745 | 3.21e-1 | 7.93e-1 |
| CN mRNA | TDP-43 | -0.3155 | 0.2650 | 2.34e-1 | 6.81e-1 |
| CN mRNA | Lewy body pathology | -0.0254 | 0.2499 | 9.19e-1 | 7.93e-1 |
| PCC mRNA | Cerebral Micro Infarcts | 0.2019 | 0.1516 | 1.83e-1 | 6.81e-1 |
| PCC mRNA | Cerebral Macro Infarcts | -0.0806 | 0.1893 | 6.70e-1 | 8.38e-1 |
| PCC mRNA | CAA | 0.1665 | 0.1319 | 2.07e-1 | 6.81e-1 |
| PCC mRNA | TDP-43 | 0.1603 | 0.2100 | 4.45e-1 | 7.93e-1 |
| PCC mRNA | Lewy body pathology | -0.1434 | 0.1960 | 4.64e-1 | 7.93e-1 |
| Boldface signifies p < 0.05. All model p values were FDR-corrected for total number of tests completed within each amyloid stratification (N=20) dlPFC Dorsolateral prefrontal cortex, CN caudate nucleus, PCC posterior cingulate cortex | | | | | |
